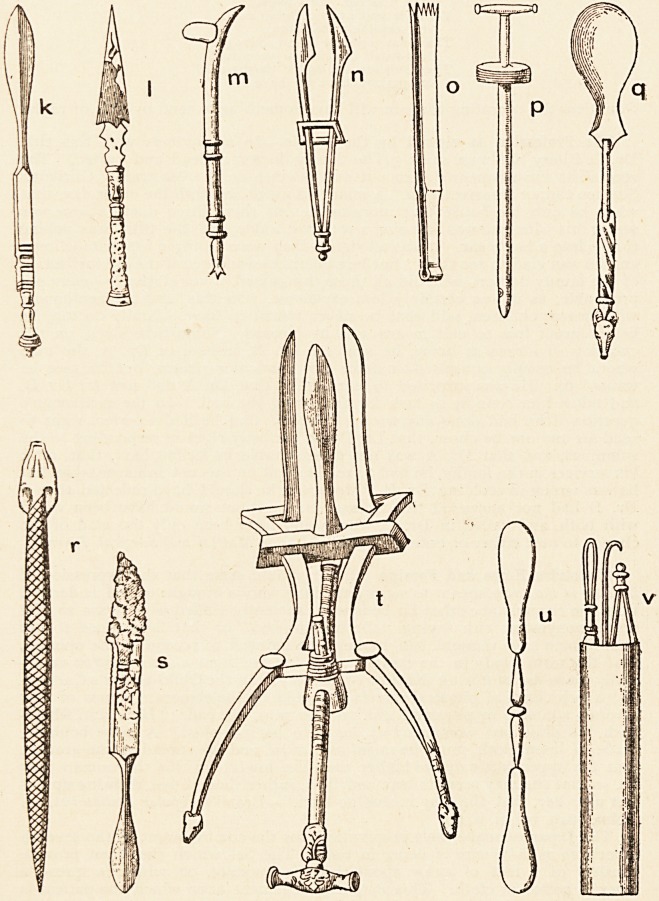# Scraps

**Published:** 1896-03

**Authors:** 


### SCRAPS

PICKED UP BY THE ASSISTANT-EDITOR.

---

**Clinical Records** (14).—Patient: "All my money cannot give me health, doctor!" Doctor (of a bygone age): "No, perhaps not; but it is of inestimable value, nevertheless. It gives your physician great confidence."

**Medical Philology** (XVII).—The word "Chitlings" used in certain ranks of society to denote parts of the intestines, mesentery, and omentum of the pig is only another form of the good Middle English word "Chitterlings," which in the *Promptorium* (1440) is met with as "Chytyrlynge, *Scrutellum, scrutum*"[1], taken by its compiler from some work by Robert Kilwardby, who was Archbishop of Canterbury from 1227 to 1278. The *Catholicon* (1483) has "a Chit*er*lyng*e*; *hilla*."[1] The word was in common use, as may be seen from the instances given by Mr. Way, Mr. Herrtage, and the *New English Dictionary*, some of which I copy. Horman in his *Vulgaria* (1519) says: "let us have trypis, chetterlyngis, and tryllybubbys ynough." Palsgrave's *Esclarcissement* (1530) gives "Chyterling, *endoile*." In Cooper's *Thesaurus* (1532) "a small gutte or chitterlyng salted" is the equivalent of "hilla." Sir Thomas Elyot in the *Castel of Helth* (1533) speaks of "the inwarde of beastes, as trypes and chytterlynges." Mr. Herrtage says that in the *Nomenclator*, which I presume is the 1548 hexaglot vocabulary mentioned by Mr. Way on p. lxxix. of the Appendix to the *Promptorium*, we find "a haggise; some call it a chitterling, some a hog's harslet." Levins, in the *Manipulus Vocabulorum* (1570), has "A chyttering, *omasum*. A chitterling, *idem*." Baret, in his *Alvearie* (1573), gives "a chitterling, *omasum;* a gut or chitterling hanged in the smoke, *hilla infumata*." In Dekker's *Honest Whore* (1604) Fustigo speaks of a meal of "Calves chaldrons, and chitterlings" (ed. Pearson, 1873, vol. ii. p. 40). In Cotgrave (1611) the English of "andouille," which is the same word as Palsgrave's "endoile," is given as "a linke, or chitterling; a big hogges gut stuffed with small guts (and other intrailes) cut into small pieces, and seasoned with pepper and salt." Cotgrave has also "*Friquenelles;* slender, & small chitterlings, or links." Sherwood's English-French addition to Cotgrave gives the French equivalent of chitterling as "le gras boyau," which Cotgrave says is called in beasts "the Inche-pinne, or Inne-pinne." In *Hudibras* (1663) will be remembered the passage (I. ii. 119-22):

His warped ear hung o'er the strings,
Which was but souse to chitterlings:
For guts, some write, ere they are sodden
Are fit for musick or for pudden.

The word was also used metaphorically. It was applied to ruffs generally, and more especially to the frill of a shirt which bore some resemblance to the mesentery. In the play *Like Will to Like* (1568), Newfangle says: "I learn'd to make ruffs like calves' chitterlings" (Dodsley's *Old English Plays*, ed. Hazlitt, vol. iii. p. 310). In Dyche and Pardon's Dictionary (1750) Chitterlings are defined as "the inwards, or hogs guts dressed for food, which are much shrivelled or curled up; from whence the cambrick ornaments worn upon the shirts of most men at this time are so called, because of their being gathered in folds and plaits." Anstey in his *New Bath Guide* (1766) mentions "a chitterlin shirt" (Letter xi.). Other instances of the word in its material and figurative senses are given in the *New English Dictionary*.

The chaldrons or chawdrons mentioned in the quotation from Dekker were also portions of the entrails of a beast. "A tiger's chaudron" was part of the ingredients of the witches' cauldron (*Macbeth*, IV. i. 33).

The etymology of chitterling and of chawdron is obscure. Chitterling may be allied to the German *Kuttel*, entrails.

1 A *Nominale* of the fifteenth century gives "*Hoc scrutum*, a trype" and "*Hec hilla*, a sawstyre" (Wright's *Vocab*. 2nd ed., 678.4 and 741.25). Sawstyre signified a sausage. Lewis and Short do not give "scrutum" or "scrutellum," but "scrutellus" is defined as "a pork-sausage." They give "hillae" as "the smaller and anterior intestines of animals (other than men and sheep)," and refer to Horace (*Sat*., II. iv. 60)—

"pernâ magis ac magis hillis
Flagitat in morsus refici,"

where the word is usually translated "sausages."

**Surgical Instruments of Antiquity.**—Under the title of "Pompeian Surgery and Surgical Instruments," Dr. N. Senn has a very interesting paper in the *Medical News* of December 28th, 1895. In June and September of last year I had, amongst the "Scraps," some notes on the instruments in the Naples Museum. By the courtesy of Messrs. Lea Brothers & Co., the publishers of the *Medical News*, I am enabled to give a reproduction, on a reduced scale, of the illustrations which accompany Dr. Senn's paper. The instruments were found in a house now known as the "Surgeon's House." This was overwhelmed when Pompeii was destroyed in the year 79. Some of the instruments show the destructive effects of the heat and oxidation. I append extracts from Dr. Senn's description of the instruments depicted: **a.** Actual cautery. Length 10 in. **b.** Bivalve speculum. Length 6 in.; width, when open, 2½ in.

**c.** Scissors. Length 4 in. **d.** Male catheter. Length $10\frac{1}{2}$ in. **e.** Hook. Length 6 in. **f.** Point of injection-syringe. Length 6 in. **g.** Forceps, each branch fitted with an engine-turned handle and a spoon-shaped blade. Length 8 in. **h.** Forceps, with serrated bite. Length $4\frac{1}{2}$ in. **i.** Cupping-glass of bronze. Height 6 in., diameter 3 in. **j.** Medicine-box with medicines, 5 in. by 3 in.

**k.** Spatula for mixing ointments. Length 7 in. **l.** Lancet for bleeding. Length 5 in. **m.** Fleam for bleeding horses. Length $5\frac{1}{2}$ in. **n.** Forceps. Length $4\frac{1}{2}$ in. **o.** Toothed dissecting-forceps. Length $7\frac{1}{2}$ in. **p.** Trocar. Length 5 in. **q.** Small spoon with bone handle. Length $5\frac{1}{2}$ in. **r.** Female catheter. Length 4 in. **s.** Bistoury. Length $5\frac{3}{4}$ in. **t.** Trivalve speculum. Length $8\frac{1}{4}$ in.; widest expansion of valves $1\frac{1}{2}$ in. **u.** Spatula. Length 7 in. **v.** Metallic case with instruments. Length 8 in. by $\frac{3}{4}$ in.

**The "Bitter Cry" of the Physician.**—A subscriber to this *Journal*, after reading a late utterance, and some recent correspondence, on Abdominal Surgery, has come to the conclusion that "the greed of the surgeon is becoming intolerable." He gives vent to his feelings by sending the following:

It may be long since surgeons first
  Took "barber" as a name;
Yet style and practice, even now,
  Are seemingly the same:
The noun replaced by adjective;
  How small the change we crave!
They still are known as "barbarous,"
  And patients have a "shave."

Doubtless the operating surgeon will have something to send by way of reply.

**The Profession as viewed by the Public.**—In an address with this title Dr. C. Ellery Stedman said: "The family doctor is dead and buried. The specialists have squeezed him out as the vines do the big trees. Christian Science cannot resurrect him. A man told me at the club the other day, that when he quit his house that morning he left there three doctors; one was seeing his wife, one was dressing a sprained ankle, and the third was poking things into a boy's ear. It was all right—they were all three first-rate fellows, and he was glad to see them; but he sometimes wondered if the old institution of the family doctor—who did all those things well, if not elegantly—were not preferable, as it was certainly less expensive. On this, another gentleman, with grown children, said that he never meant to have a family doctor; he held himself free to call in any one he pleased. Some time since, on the occasion of illness at home, he sent for Dr. B, instead of Dr. A, the man whom he usually consulted—not for any particular reason, but because he wanted to. He was surprised to learn, later, that Dr. A had sent for Dr. B, and taken him soundly to task for answering the call. To the gentleman's question if he had done any wrong, I replied that he had a perfect right to send for any one he chose, that Dr. B was perfectly right in responding to the summons, and that Dr. A was not unreasonable in feeling hurt, that, after his services in the family, he had to realize that he was not indispensable, but he was wrong in scolding Dr. B. Moreover, he should have reflected that if Dr. B had not answered the message, the patient would have been vexed with both, and called in Dr. C, who would have been only too glad of the chance to oust either or both of them."—*Boston Medical and Surgical Journal.*

**Obstetrics Home and Foreign** (1).—It should seem that the expression of *the lady in the straw*, meant to signify the lady who is brought to bed, is derived from the circumstance that all beds were anciently stuffed with straw, so that it is synonymous with saying, "the lady in bed," or that is confined to her bed. Some have thought, but I cannot be induced to accede to the opinion, that the term "lady in the straw" takes its rise from *a straw mattress* necessarily made use of during the time of delivery. In the Child-bearer's Cabinet, in "a rich closet of physical secrets collected by the elaborate paines of four severall students in physick," 4to. no date, p. 9, we read: "How, and wherewith the child-bed woman's bed ought to be furnished. A large boulster, made of linen cloth, must be *stuffed with straw*, and be spread on the ground, that the upper part may lye higher than the lower; on this the woman may lie, so that she may seem to lean and bow, rather than to lye, drawing up her feet unto her, that she may receve no hurt."—Brand's *Popular Antiquities*, ed. Bohn, 1849, vol. ii. p. 66.

The Japanese make their preparations for the coming event in the seventh month, so as to be sure of being in time. The bed which they then provide consists of a mat of straw about three feet square, on which is spread a layer of cotton or cloth. This simple arrangement upon which the patient is to be delivered is then set aside to be available at any emergency. At the time of labour this mat is arranged so that the woman may sit on it with her legs under her thighs. A portion of the mat is bent to form a back for her, and is supported by cushions. The sitting position is retained for about three days after the birth of the child.—Engelmann's *Labor among Primitive Peoples*, 1882, pp. 38, 129, 178.

## Figures and Tables

**Figure f1:**
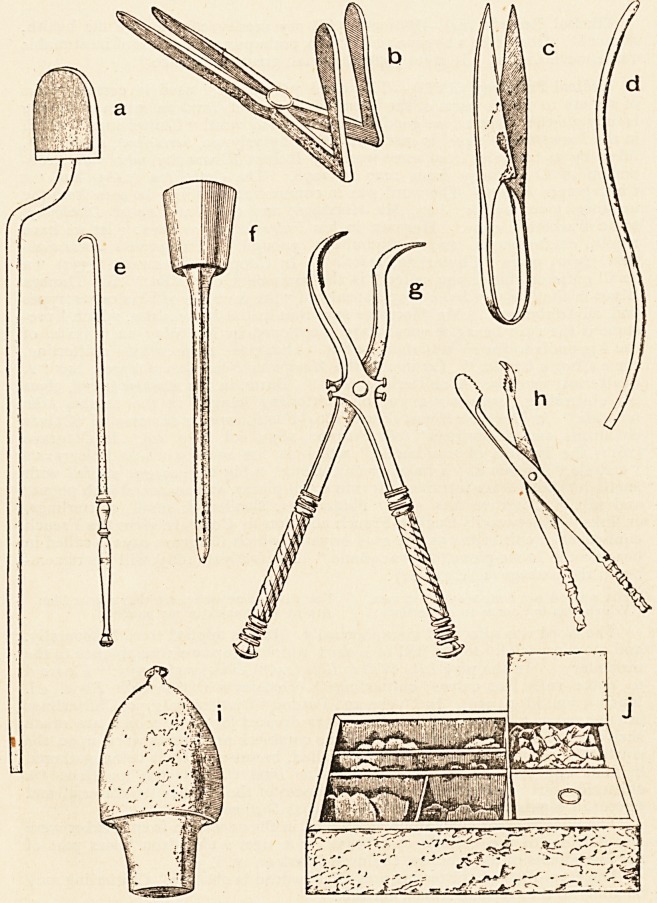


**Figure f2:**